# Rhabdomyosarcoma in Adults: De Novo or Conversion From Non-seminomas?

**DOI:** 10.7759/cureus.55449

**Published:** 2024-03-03

**Authors:** Moutaz Ghrewati, Anas Mahmoud, Tala Beilani, Mehandar Kumar

**Affiliations:** 1 Oncology, St. Joseph’s University Medical Center, Paterson, USA; 2 Internal Medicine, St. Joseph’s University Medical Center, Paterson, USA; 3 Oncology, Kansas City University, Kansas City, USA

**Keywords:** afp, b-hcg, cancer, germ cell tumor, ldh, non-seminoma, pure embryonal carcinoma, rhabdomyosarcoma, seminoma, testicular cancer

## Abstract

Rhabdomyosarcoma (RMS) is a highly sporadic, very aggressive, and fatal soft tissue tumor in adults. Although more common and treatable in the pediatric population, the occurrence of pleomorphic RMS in adults has a low incidence. Hence, it is not easy to treat. Surgery is the primary definitive treatment, along with radiation therapy, while adjuvant chemotherapy has recently gained popularity. We present an infrequent case of RMS in a patient with a recent history of mixed non-seminomatous germ-cell tumor testicular cancer. Therefore, it was challenging to treat the RMS as a new malignancy or as a recurrence of non-seminomatous testicular cancer. Our patient passed away, unfortunately, but we hope this case can help the minimal data in this regard in order to save more lives in the future.

## Introduction

Germ cell tumors can be either seminomas (more common) or non-seminomas. Non-seminomas are more likely to cause metastases, typically in the lungs and liver, than seminomas [1]. Although active surveillance with serum tumor markers and computerized tomography help detect early recurrence, there is still some chance to be missed [2], which was the case in our patient. We present a patient with a non-seminomatous testicular germ cell tumor who was fully cured and had gone through active surveillance for two years; however, he presented back with pleomorphic rhabdomyosarcoma (RMS). Despite discussing his rare case with significant institutions and receiving chemotherapy, he unfortunately passed away.

## Case presentation

**Figure 1:**
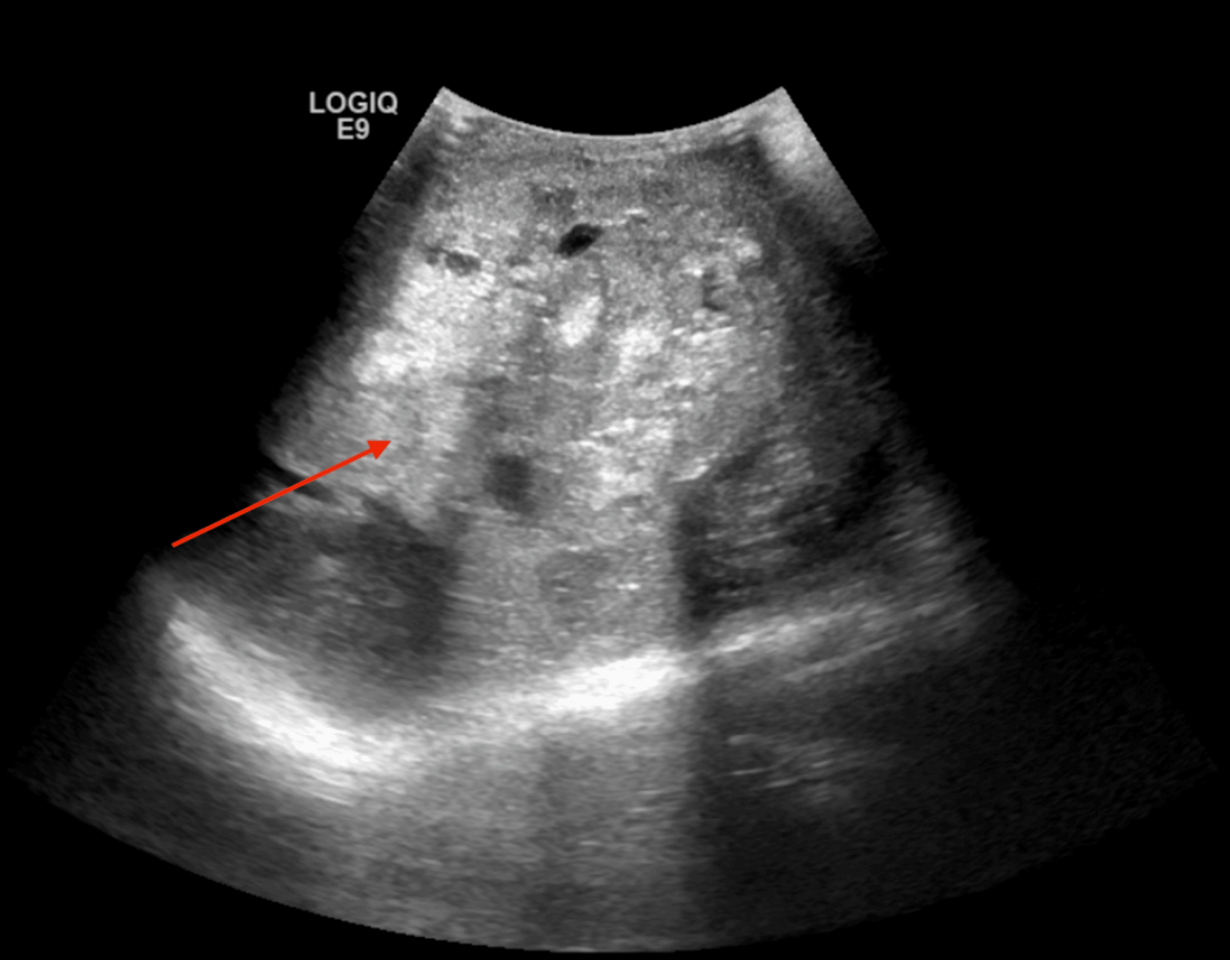
Ultrasonography (US) of the scrotum showing a large mass in the left testicle distending the left scrotum and containing internal vascularity, calcifications, and necrosis, suspicious of a malignant mass (red arrow).

**Figure 2:**
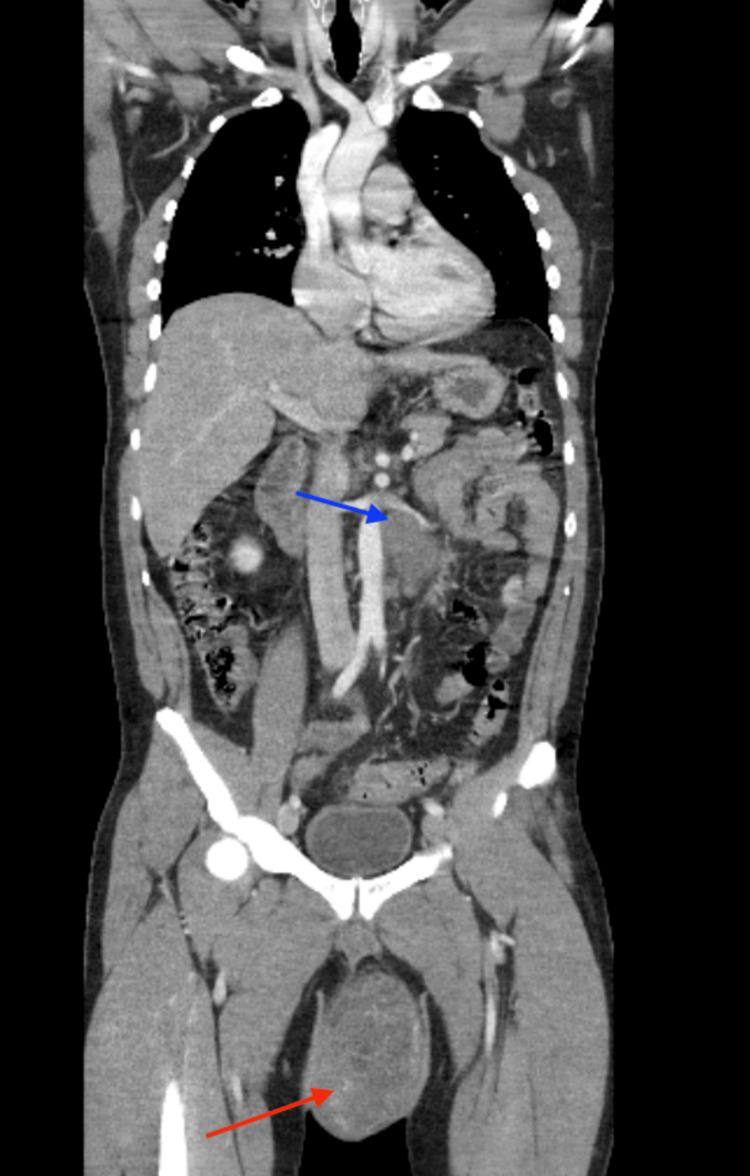
Computed tomography (CT) of the chest, abdomen, and pelvis showing a large scrotal mass (red arrow) with para-aortic lymph node metastases (blue arrow).

**Figure 3:**
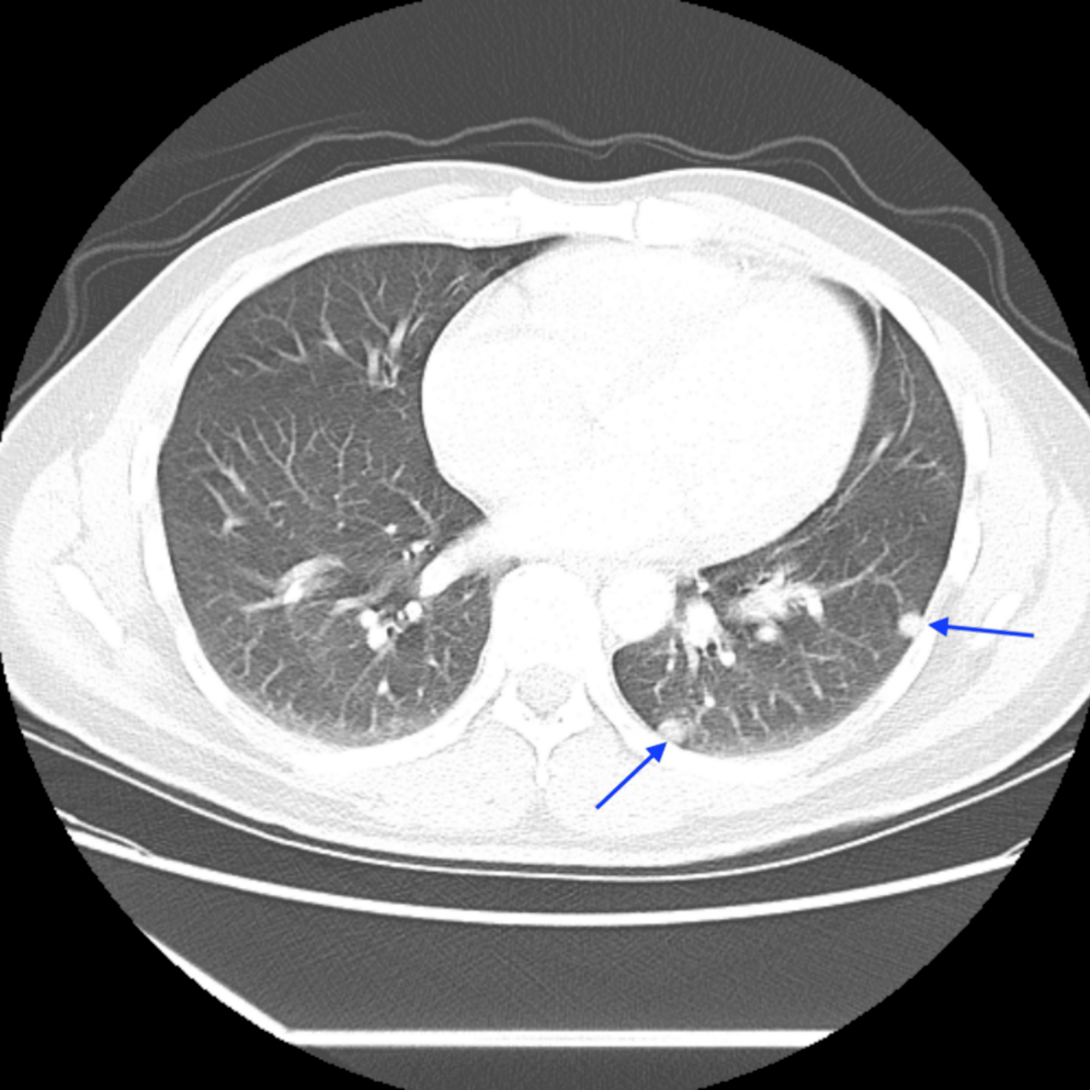
Computed tomography (CT) scan of the chest showing metastases (blue arrows)

We present a 39-year-old Hispanic male, with no past medical history, who presented with complaints of a left testicular mass that he noticed eight months ago, which has been progressively getting larger in size. The patient also complained of recent unintentional 4-pound weight loss and night sweats. Vitals were stable. Physical examination revealed a testicular mass that measured 12 x 12 cm, which was nontender and not warm to touch. Ultrasonography (US) of the scrotum showed a large mass in the left testicle distending the left scrotum and containing internal vascularity, calcifications, and necrosis, suspicious of a malignant mass (Figure 1). CT scan of the chest, abdomen, and pelvis with contrast showed a large scrotal mass with para-aortic lymph node metastases and lung metastases (Figures 2, 3). Blood work was pertinent for elevated levels of alpha-fetoprotein (AFP) 4548, beta human chorionic gonadotropin (B-HCG) 431, and lactate dehydrogenase (LDH) 593. The patient was staged as IIIb (CT4cn3m1a), and his Eastern Cooperative Oncology Group (ECOG) was 0-1.

**Figure 4:**
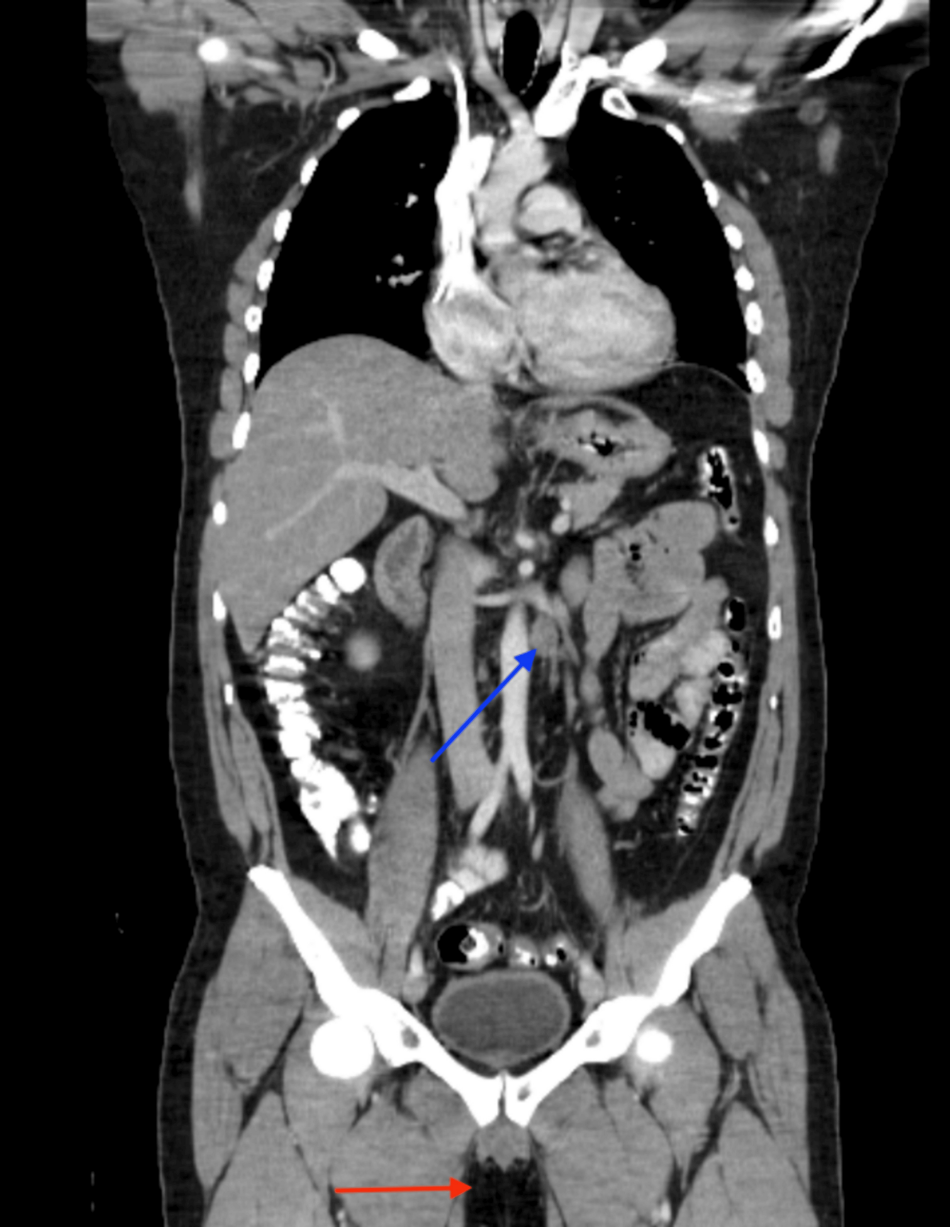
Computed tomography (CT) scan of the chest, abdomen, and pelvis showing improvement (red arrow with no testicle and blue arrow showing a decrease in the para-aortic lymph nodes).

The urologist performed a radical inguinal orchiectomy, and the pathology was sent. After surgery, repeat labs showed LDH 465, B-HCG 161, and AFP 5635. The pathology came back and demonstrated mixed germ cell tumor, non-seminoma (yolk sac with embryonal and teratoma). Subsequently, the patient received four cycles of bleomycin, etoposide, and cisplatin/platinum (BEP). After four cycles of BEP, the repeat levels trended down significantly (LDH 139, B-HCG <5, and AFP 3.8). CT scan of the chest, abdomen, and pelvis showed a decrease in the size of para-aortic lymph nodes (Figure 4).

**Figure 5:**
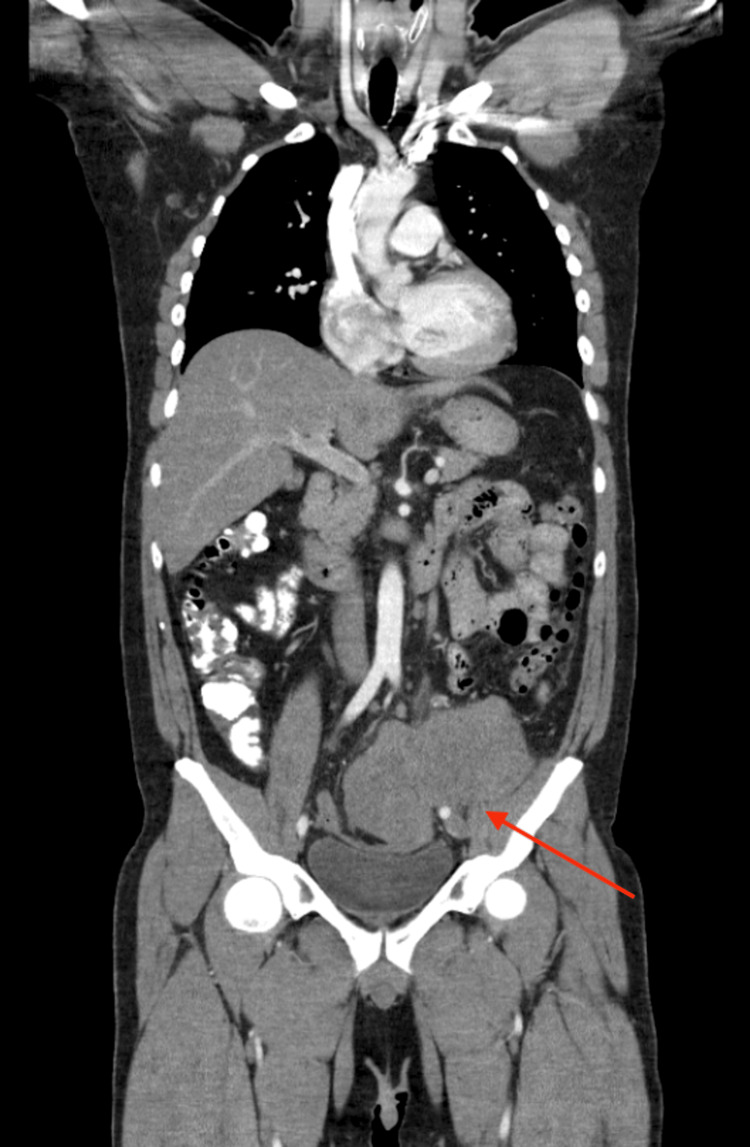
Computed tomography (CT) scan of the chest, abdomen, and pelvis showing a lobulated heterogeneous soft tissue mass in the left lower quadrant in the abdomen, measuring approximately 10 x 13 cm (red arrow])

The patient remained on surveillance (office visits every two months, tumor markers every two months, and CT scan every six months), which confirmed complete response for two years, until the patient presented later with complaints of severe left lower-quadrant abdominal pain associated with severe burning on micturition. CT scan of the chest, abdomen, and pelvis with contrast showed a lobulated heterogeneous soft tissue mass in the left lower quadrant in the abdomen, measuring approximately 10 x 13 cm, leading to left hydroureteronephrosis due to obstruction of the distal left ureter, along with notable metastatic retroperitoneal lymphadenopathy (Figure 5). US of the scrotum did not show any concerning masses. Tumor markers were AFP 2, B-HCG <1, and LDH 2676. A mass biopsy was sent to the pathology lab with a prelim report suggestive of the recurrence of the germ cell tumor, and the oncology team decided to start chemotherapy with vinblastine, ifosfamide, and cisplatin (VeIP), along with Mensa to protect against hemorrhagic cystitis.

Soon after completing the first cycle, the pathology results revealed a poorly differentiated malignant neoplasm, consistent with embryonal RMS (somatic-type malignancy), which is most likely arising or transformed from the previous germ cell tumor. The case was then sent to a testicular cancer specialist; meanwhile, the patient received the second dose of VeIP, which resulted in an improvement in tumor markers. Repeat tumor markers were AFP 2, B-HCG <1, and LDH 373. The summary of tumor markers is shown in Table 1. 

**Table 1:** Summary of tumor markers since the first admission, after surgery, after receiving chemotherapy, and after representation with rhabdomyosarcoma

	Normal range values	Admission	After surgery	After chemotherapy	After rhabdomyosarcoma
Alpha-fetoprotein (AFP)	0-40 ng/mL	4548 ng/mL	5635 ng/mL	3.8 ng/mL	2 ng/mL
Beta human chorionic gonadotropin (B-HCG)	Less than 2 mIU/mL	431 mIU/mL	161 mIU/mL	<5 mIU/mL	<1 mIU/mL
Lactate dehydrogenase (LDH)	140-280 U/L	593 U/L	465 U/L	139 U/L	373 U/L

**Figure 6:**
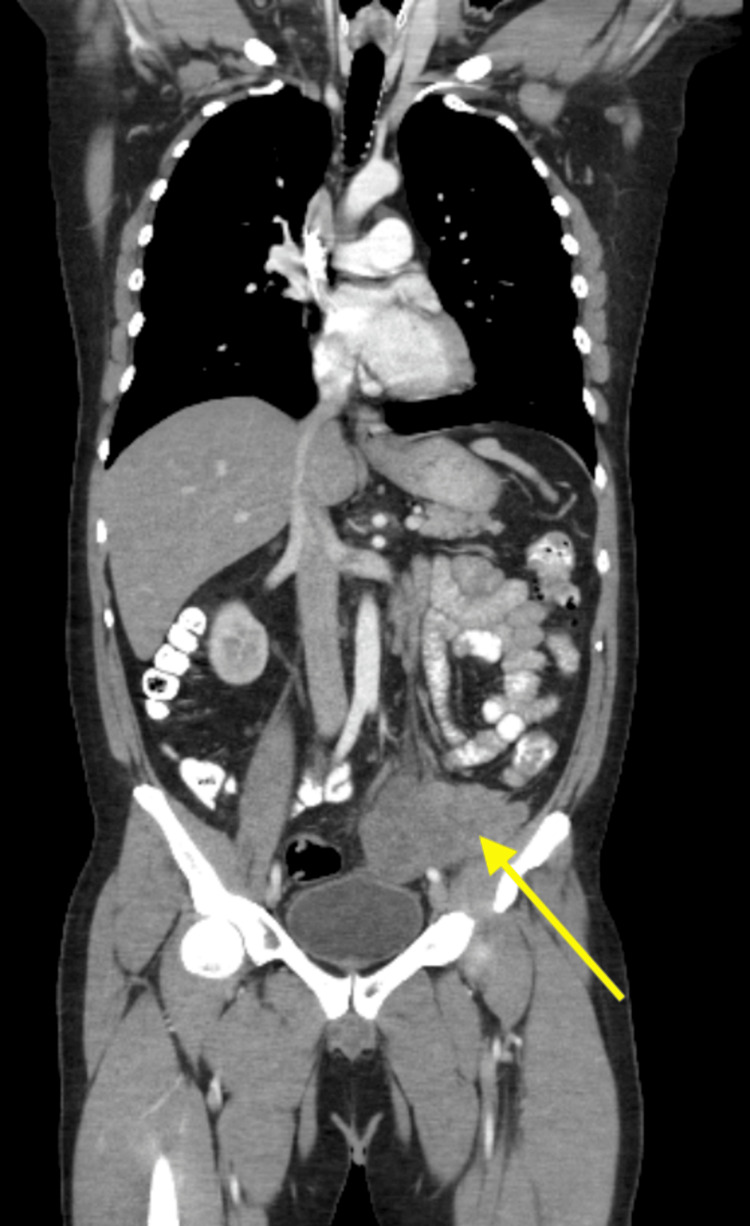
CT scan of the chest, abdomen, and pelvis showing interval decrease in the size of left pelvic mass (yellow arrow)

The case was presented at the tumor board, and the decision was made to treat this unusual malignancy as RMS with the suggested regimen of VCD/IE regime and perform a total resection of the mass. Moreover, after two cycles of VCD/IE, a CT scan of the chest, abdomen, and pelvis showed an interval decrease in the size of the left pelvic mass with improving left-sided hydroureteronephrosis (Figure 6). The patient underwent laparotomy, which showed tumor deposits along the momentum and peritoneum, which were removed to enter the peritoneum. After entering, extensive tumor deposits were seen along the bilateral sidewalls of the abdomen, retroperitoneum, pelvis, peritoneum, and omentum. The sigmoid colon was noted to be densely adherent and encased by the retroperitoneal pelvic mass, and then an end sigmoid colostomy was made. Even after the conclusion of debulking, there were still noted tumor deposits along the lateral abdominal sidewalls, omentum, and retroperitoneum. The pathology report showed RMS in the peritoneum, omentum rectum, intra-abdominal mass with involvement of peri-colonic tissue, and lymphovascular and perineural invasion without affecting the colonic mucosa.

**Figure 7:**
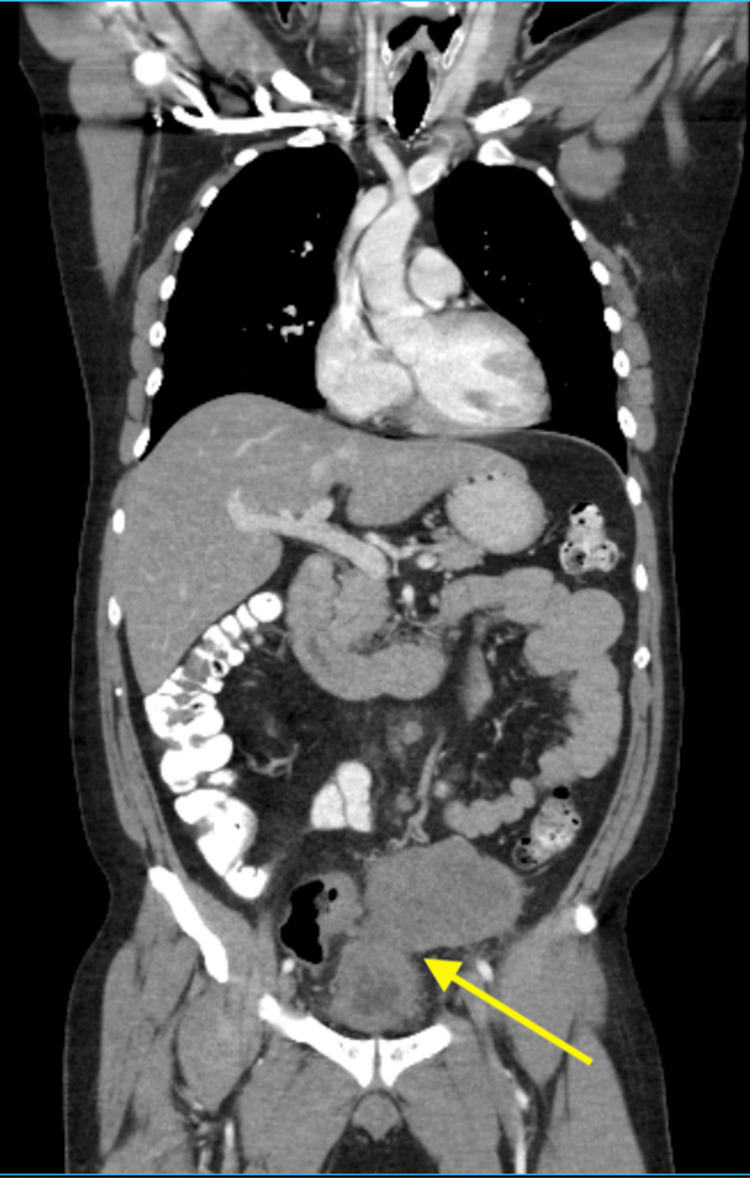
CT scan of chest, abdomen, and pelvic showing invasion of the rhabdomyosarcoma into the urinary bladder (yellow arrow)

After the operation, the patient was placed in the intensive care unit, and unfortunately, a repeat CT scan of the abdomen showed further progression of disease and invasion of the bladder (Figure 7). The patient kept on declining, and after extensive discussion with the patient and his family, the decision was made to proceed with comfort measures, especially after the ECOG score dropped to 4. Soon after, the patient passed away, unfortunately.

## Discussion

Testicular cancers are the most common solid tumors in men aged 15-35 years; however, they only account for 1% of all cancers diagnosed in the US [3]. Fortunately, testicular cancer represents a curable cancer in most cases due to sensitivity to chemotherapy. Testicular cancers are classified into germ cell tumors and non-germ-cell tumors. Germ cell tumors, which arise from the germ cells producing sperms, account for 95% of all testicular cancers [1,2,3]. Germ cell tumors are further classified into two main categories: seminomas and non-seminomas. This classification is essential to formulate a treatment plan. Seminomas usually carry a better prognosis as the cancer cells tend to metastasize late, and they are both chemosensitive and radiosensitive [1,3]. On the other hand, non-seminomas are usually diagnosed at an advanced stage, and specific subtypes are prone to be radioresistant, which makes the prognosis less favorable. Non-seminomas are further subclassified into four main categories: embryonal carcinoma, choriocarcinoma, teratoma, and yolk cell carcinomas [3]. Despite a few differences between these subtypes, most non-seminomas tend to have a mixed histology. Embryonal carcinomas are very painful, and they look like a hemorrhagic mass with necrosis under the microscope. They display more comprehensive ranges of tumor markers, including B-HCG and AFP [1,2,3,4]. The teratoma subtype carries a variable prognosis depending on the age and sex groups, as it is more likely to be malignant in adult males but benign in children and females. The yolk sac subtype, also called endodermal sinus tumor, usually has a yellow mass or mucinous, and Schiller Duval bodies are characteristic findings under the microscope [1]. AFP is consistently elevated, unlike the embryonal subtype. The yolk sac subtype is the most common testicular cancer in children under the age of three years. Choriocarcinoma has a characteristic hematogenous metastasis to the lungs and the brain. Due to elevated B-HCG, choriocarcinomas can cause gynecomastia and symptoms of hyperthyroidism [3,4]. 

The initial step of evaluating a testicular mass is done by scrotal ultrasound and obtaining baseline tumor markers (AFP, B-HCG, and LDH) [4]. Definitive diagnosis is made through radical inguinal orchiectomy and pathology. Needle biopsy is contraindicated as the tumor might seed and cause dissemination. After confirming the diagnosis, a chest X-ray and CT scan of the chest, abdomen, and pelvis should follow to evaluate metastases. Repeat levels of tumor markers after orchiectomy must occur to confirm eradication and ensure cancer-free status. It might take a long time for tumor markers to fall. Sperm cryopreservation should be offered prior to orchiectomy. Management of testicular cancers after orchiectomy depends on the histology, staging, and the presence or absence of tumor markers. Staging with post-orchiectomy tumor markers helps risk-stratify non-seminomas into good, intermediate, and high-risk [5]. Treatment modalities include orchiectomy with or without retroperitoneal lymph node dissection (RPLND) and chemotherapy [6]. As germ cells tend to arise from sperm-producing cells, they rarely convert into other malignancies. Despite successful treatment, the risk for recurrence and developing another solid tumor remains, which was, unfortunately, the case in our patient who developed RMS. The de novo RMS has a scarce incidence, and its transformation is even scarcer. Therefore, consulting with influential centers is warranted [7]. 

Sarcomas are uncommon malignant soft tissue tumors, representing 0.8% of all cancers in the United States [8]. RMS, a rare subtype of sarcomas, has a mesenchymal origin, is very aggressive, and has a high metastatic potential. RMS comprises striated muscle cells that have failed to differentiate fully. It is most commonly seen in the pediatric population and extremely rare in adults; therefore, studying this disease in adults is challenging. Embryonal RMS is the most common subtype of RMS in children, whereas pleomorphic RMS is usually found in adults. Our case was rare because the pathology showed embryonal RMS in an old patient. RMS is extremely rare in the abdomen and pelvis [9]. Whole-body MRI may offer a clear demonstration of soft tissue metastases. The Intergroup Rhabdomyosarcoma Studies (IRS) recommends using children's multimodal therapy (MMT), including resection, chemotherapy, and radiation, for adults to eliminate microscopic and macroscopic residual tumors [10]. Adjuvant chemotherapy with doxorubicin-based regimes and ifosfamide showed a reduction in recurrence rate and improved prognosis in patients with localized resectable soft-tissue sarcoma [11]. Age, tumor size (>5 cm), stage, and nodal involvement determine the prognosis and the odds of relapse. Small studies have found that in adults with RMS, chemotherapy responders had improved metastasis-free survival compared to nonresponders. A large study including 235 pediatric and adolescent patients with para-testicular RMS concluded that RPLND improved overall survival among patients older than 10 years [12]. Our patient did not undergo RPLND as the repeat CT scan after chemotherapy showed complete resolution of lymph node metastases. Our unique case highlights the possibility and the rarity of non-seminoma conversion to RMS, which could help guide the expectations of treatment and the prognosis of such a rare incidence.

## Conclusions

RMS is a typical tumor in childhood and adolescence and is very rare to occur in adults. Although its treatment in children has seen significant advances and success, this did not translate into similar success in adults due to the scarcity among adults. On the other hand, germ cell tumors in adults are curable. With appropriate staging, surgery and adjuvant chemotherapy have revolutionized the successful treatment. With close surveillance, the relapse rate can be minimal; however, it is still possible. Our case represents a unique relapse of a non-seminomatous germ cell tumor despite close surveillance into a pleomorphic RMS that was resistant to treatment, and the patient passed away.
